# Supplementary material for: Unsupervised learning of perceptual feature combinations
Source: PLoS Comput Biol. 2024 Mar 5;20(3):e1011926. doi: 10.1371/journal.pcbi.1011926 (PMC10942261; doi:10.1371/journal.pcbi.1011926)
Supplement: S1 Appendix — (PDF) [file pcbi.1011926.s001.pdf]

S1 Appendix: Additional analyzes of reference methods

Provided for the paper: “Unsupervised learning of perceptual feature combinations”
Minija Tamosiunaite, Christian Tetzlaff, Florentin Wörgötter

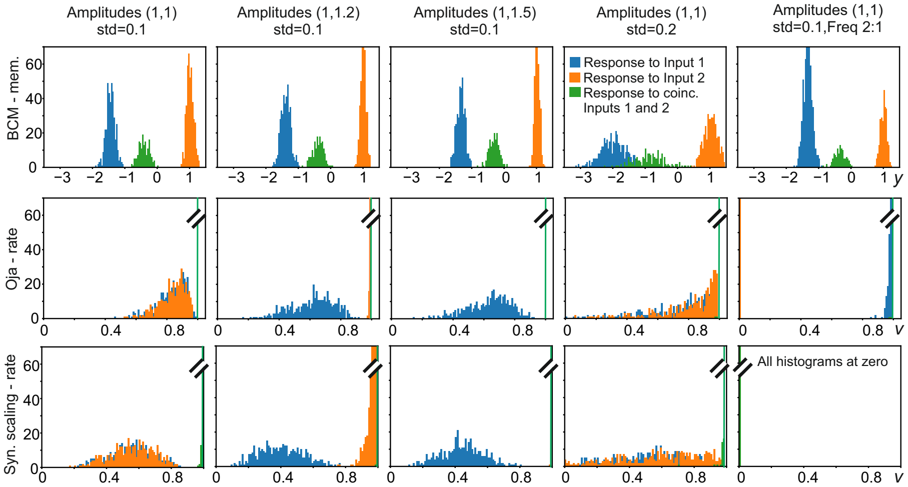

Fig. S1.1. Unsatisfactory cases for the reference methods: BCM, Oja and synaptic scaling using linear (y, marked *mem*) or non-linear (v, *rate*) output terms in the learning. Input coincidence is 30% everywhere. Amplitudes and STD shown above each column. For BCM (linear) $\boldsymbol{\omega}(0) = [0.001, 0.001]^T$, for Oja and synaptic scaling (non-linear): $\boldsymbol{\omega}(0) = [0.2, 0.2]^T$. Other parameters: BCM linear: $v_0 = 1$, $\gamma = 10$, $\Theta_M(0) = 0.2$; Synaptic Scaling: $v_0 = -1.35$, $\nu = 0.7$, for all $\mu = 0.001$.

Here we show that the classical BCM-rule [1] for a 2-input system of the type we are analyzing in the current study cannot be satisfactorily applied in the *linear* case. While fixed points for the synaptic weights exist, one of the weights is always negative. Thus, this leads to unrealistic results (see the top row in figure S1.1, labeled according to conventions of our manuscript “Membrane BCM” (BCM-mem)). On the other hand, Oja’s method [2] as well as synaptic scaling [3] cannot be satisfactorily applied after *nonlinear* output transform as given by eq. (2) in the main text. For results see lines 2 and 3: “Oja - rate” and “Syn. scaling - rate” in Figure S1.1.

References

1. Bienenstock EL, Cooper LN, Munro PW. Theory for the development of neuron selectivity: orientation specificity and binocular interaction in visual cortex. Journal of Neuroscience. 1982;2(1):32–48.

2. Oja E. Simplified neuron model as a principal component analyzer. Journal of mathematical biology. 1982;15:267–273.

3. Tetzlaff C, Kolodziejski C, Timme M, Wörgötter F. Synaptic scaling in combination with many generic plasticity mechanisms stabilizes circuit connectivity. Frontiers in Computational Neuroscience. 2011;5:47. doi:10.3389/fncom.2011.000.
