# Supplementary material for: Unsupervised learning of perceptual feature combinations
Source: PLoS Comput Biol. 2024 Mar 5;20(3):e1011926. doi: 10.1371/journal.pcbi.1011926 (PMC10942261; doi:10.1371/journal.pcbi.1011926)
Supplement: S2 Appendix — (PDF) [file pcbi.1011926.s002.pdf]

S2 Appendix: Parameter analysis for the BCM rule in case of two inputs.

Provided for the paper: "Unsupervised learning of perceptual feature combinations"

Minija Tamosiunaite, Christian Tetzlaff, Florentin Wörgötter

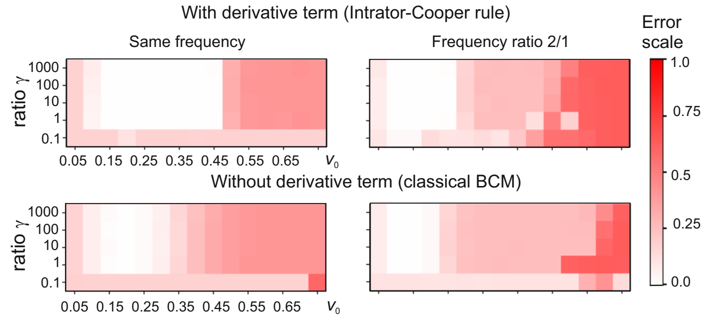

Fig. S2.1. Classification error (coincidence vs. not coincidence on a neuron with two inputs) of the BCM-rule in respect to parameter variations. Parameters are target output v_0 and ratio γ, between time constants of two differential equations. Decision threshold is 0.5. Averages over 20 trials are shown. Initial weights are $\boldsymbol{\omega}(0) = [0.2, 0.2]^T$, $\Theta_M(0) = 0.2$; learning rate $\mu_0 = 0.001$; Euler integration with step $dt = 1$. Intrator-Cooper version is provided in the top row and classical BCM in the bottom row. White areas indicate favourable parameter settings.

Here we provide a parameter analysis for BCM rules: classical BCM, implementation based on [1], and Intrator-Cooper version, implemented based on [2], see method section for more details. Errors are marked in shades of red. White areas mean error-free areas, and thus appropriate parameter combinations. It is visible that white areas are wider in the Intrator-Cooper version, upper row. Also, white areas are wider, when presentation frequencies of both inputs are equal, as compared one input twice more frequent (first vs. second column). While a correct choice of parameter v_0 is proven to be important, ratio γ is not very relevant for coincidence separation.

References

1. Toyoizumi T, Kaneko M, Stryker MP, Miller KD. Modeling the dynamic interaction of Hebbian and homeostatic plasticity. Neuron. 2014;84(2):497–510. doi:10.1016/j.neuron.2014.09.036.

2. Blais BS, Cooper L. BCM theory. Scholarpedia. 2008;3(3):1570. doi:10.4249/scholarpedia.1570.
