# Supplementary material for: Unsupervised learning of perceptual feature combinations
Source: PLoS Comput Biol. 2024 Mar 5;20(3):e1011926. doi: 10.1371/journal.pcbi.1011926 (PMC10942261; doi:10.1371/journal.pcbi.1011926)
Supplement: S1 Code Repository — (ZIP) [file pcbi.1011926.s003.zip › code_for_figures/readme_condensed_version.docx]

This is the code for the paper „Unsupervised learning of perceptual feature combinations” by Minija Tamosiunaite, Christian Tetzlaff and Florentin Wörgötter.

The code is extensively commented and further in this readme it is explained how to use the code figure-wise. However, one code piece many times represents several plots and one hast to enter corresponding parameter values (input amplitudes, STDs, method parameters) as given in figure legends in the paper.

The code is mostly in Python, except one piece in Octave.

Some code in Python requires Numba: <https://numba.pydata.org/>. Codes will run without that, too, however it takes some 20 times longer. When running without Numba, please, erase the lines @nb.jit(nopython=True)

There is an „Imports.py“ script (slightly over-complete) which is needed, however, for all Python scripts.

**Figure 1** is for demonstration purposes only. It could potentially be obtained using code

ALL_rule_two_inputs_histograms_Figs_4B_and_5.py using high learning rate, i.e. hebb_learn_rate=0.01

**Figure 2** is a straightforward plot of equations 2 and 5, thus no code provided.

**Figure 3** is a conceptual drawing.

**Figure 4:**

**A)** Octave code: AMH_and_ALL_analytical_solution_Fig_4A.m

**B)** First row: ALL_rule_two_inputs_histograms_Figs_4B_and_5.py

Second row: AMH_rule_two_inputs_histograms_Fig_4B.py

**Figure 5:**

ALL_rule_two_inputs_histograms_Figs_4B_and_5.py

**Figure 6:**

Please, run in a sequence:

ALL_error_plot_calculation_Fig_6.py

ALL_error_plotting_Fig_6.py

**Figure 7:**

First row: BCM_histograms_Fig_7.py

Second row: Oja_histograms_Fig_7.py

Third row: Syn_scaling_histograms_Fig_7.py

**Figure 8:**

**A)** For ALL rule: ALL_rule_three_inputs_histograms_Figs_8A_and_9A.py

For BCM rule: BCM_histograms_3inputs_Figure8A.py

**B)** For ALL rule, first row: run in a sequence:

ALL_error_plots_3inputs_calculation_Fig_8B.py

ALL_error_3inputs_ploting_Fig_8B.py

For BCM rule, second row: run in a sequence:

BCM_error_3inputs_calculation_Fig_8B.py

BCM_error_3inputs_plotting_Fig_8B.py

**Figure 9:**

**A)** Row 1, error plots: Run in a sequence:

ALL_error_plots_3inputs_calculation_Fig_8B.py

ALL_error_3inputs_ploting_Fig_8B.py

Row 2, example histograms:

ALL_rule_three_inputs_histograms_Figs_8A_and_9A.py

**B)** Left, ALL rule: ALL_rule_barplot_5inputs_Figure9B.py

Right, BCM rule: BCM_barplot_5inputs_Figure_9B.py

**Figure 10:**

Left, three inputs: Run a sequence:

command_for_3input_graph_Figure_10_and_11.py (uses graph_3inputs_Figs_10_and_11.py)

combination_analysis_3inputs_graph_Figure_10_11.py

Plot “three_set.pdf” will appear in the working directory

Right, five inputs: Run a sequence:

command_for_5input_graph_Figures_10_and_11.py (uses graph_5inputs_Figs_10_and_11.py)

combination_analysis_5inputs_Figures_10_and_11.py

Plot “thrsets.pdf” will appear in the working directory

**Figure 11:**

**A and B)** Three inputs: Run a sequence:

command_for_3input_graph_Figure_10_and_11.py (uses graph_3inputs_Figs_10_and_11.py)

combination_analysis_3inputs_graph_Figure_10_11.py

Change evaluation threshold thr_eval=0.7 in line 61 as required

Printout of the one before last line is put on excel for different cases and makes the figure

C and D) Five inputs: Run a sequence

command_for_5input_graph_Figures_10_and_11.py (uses graph_5inputs_Figs_10_and_11.py)

combination_analysis_5inputs_Figures_10_and_11.py

Change in line 88 which threshold to use: pl=copy.deepcopy(pl_07) for threshold 0.7

Printout of the one before last line is put on excel for different cases and makes the figure

**Figure 12:**

Left, three inputs: run in a sequence:

command_for_3input_graph_Figure_12.py (uses graph_3inputs_Fig_12.py)

Change in line 16, how much times to increase weights: times_weight_increase=2

combination_analysis_3inputs_graph_Figure_12.py

Change evaluation threshold thr_eval=0.7 in line 61 as required

Right, five inputs: Execute a sequence:

command_for_5input_graph_Figure_12.py (uses graph_5inputs_baseline_Figure_12.py)

Change in line 56, how much times to increase weights: times_weight_increase=2

combination_analysis_5inputs_Figure_12.py

Change in line 89 which threshold to use: pl=copy.deepcopy(pl_07) (threshold 0.7)

Read out results on means from one before the last readout of inputs

**Figure 13:**

Run in a sequence:

command_for_5input_graph_Figure_13.py (uses graph_5inputs_inhibition_Figure_13.py)

combination_analysis_5inputs_Figures_13.py

Figure thrsets.pdf is in the paper

**Figure S2.1:**

Run in a sequence:

BCM_error_2inputs_calculation_Fig_14.py

BCM_error_2inputs_plotting_Fig_14.py

For Intrator-Cooper version: Line 133 needs be commented out, line 132 shall be
 un-commented

For Classical BCM: Line 133 shall be un-commented, line 132 shall be commented

Figure “errors.pdf” is given in the paper.
