# Supplementary material for: Unsupervised learning of perceptual feature combinations
Source: PLoS Comput Biol. 2024 Mar 5;20(3):e1011926. doi: 10.1371/journal.pcbi.1011926 (PMC10942261; doi:10.1371/journal.pcbi.1011926)
Supplement: S1 Code Repository — (ZIP) [file pcbi.1011926.s003.zip › code_for_figures/readme_extended_version_w_parameter_changes_for_pannels.pdf]

This is the code for the paper „Unsupervised learning of perceptual feature combinations" by Minija Tamosiunaite, Christian Tetzlaff and Florentin Wörgötter.

The code is extensively commented and further in this readme it is explained how to use the code figure-wise. However, one code piece many times represents several plots and one hast to enter corresponding parameter values (input amplitudes, STDs, method parameters) as given in figure legends in the paper. What parameters to add for each concrete panel is detailed for each figure below.

The code is mostly in Python, except one piece in Octave.

Some code in Python requires Numba: https://numba.pydata.org/. Codes will run without that, too, however it takes some 20 times longer. When running without Numba, please, erase the lines @nb.jit(nopython=True)

There is an „Imports.py" script (slightly over-complete) which is needed, however, for all Python scripts.

Figure 1 is for demonstration purposes only. It could potentially be obtained using code

ALL_rule_two_inputs_histograms_Figs_4B_and_5.py using high learning rate, i.e. hebb_learn_rate=0.01

Figure 2 is a straightforward plot of equations 2 and 5, thus no code provided.

Figure 3 is a conceptual drawing.

Figure 4:

A) Octave code: AMH_and_ALL_analytical_solution_Fig_4A.m

For different panels:

- Change annealing threshold v_a called thr in line 4
- Change Factor Phi called niu in line 6
- Readout in lines 22 and 39

B) First row: ALL_rule_two_inputs_histograms_Figs_4B_and_5.py

For different panels:

- Comment line 31, but uncomment 32 and comment line 51, but uncomment 52, to set 10% overlap, all panels
- Change std for two inputs (both equal were used in the panels] in lines 47 and 64
- Change factor Phi=size in line 49
- Set niu called anneal in line 67 to value 0.2
- Histograms provided in the manuscript are plotted in lines 201:203

Second row: AMH_rule_two_inputs_histograms_Fig_4B.py

For different panels:

- Change std for two inputs (both equal were used in the panels] in lines 47 and 64
- Change factor Phi=size in line 49
- Histograms provided in the manuscript are plotted in lines 201:203

Figure 5:

ALL_rule_two_inputs_histograms_Figs_4B_and_5.py

For different panels:

- Set amplitude factor called size=1 in line 49 to get the first row in panel A and first two images in panel B
- Set amplitude factor called size=1.2 in line 49 to get the second row in panel A
- Set amplitude factor called size=1.5 in line 49 to get the third row in panel A
- Set amplitude factor called size=1 in line 49 to get the first two images in panel B
- Set amplitude factor called size=1.2 in line 49 to get the third image in panel B
- Set amplitude factor called size=1.1 in line 49 and set factor size1=1.2 in line 46 to get the fourth image in panel B
- Keep the setting of parameters freq1, freq2 in lines 31 and 51 as they are, to obtain column 3 in Panel A (30% overlap)
- Change the setting of freq1 and freq2 in lines 31 and 51 for the second column in Part A and part B (50% overlap) to:
    - freq1= 0.5+0.1667
    - freq2=1-0.5-0.1667
- Comment line 31, but uncomment 32 and comment line 51, but uncomment 52, to set 10% overlap
- Histograms provided in the manuscript are plotted in lines 201-203

Figure 6:

Please, run in a sequence:

ALL_error_plot_calculation_Fig_6.py

ALL_error_plotting_Fig_6.py

For different panels in “calculation” program ALL_error_plot_calculation_Fig_6.py:

- For 30% coincidence, case of equal frequency, use: freq1=0.5+0.0882 in line 96 and freq2=0.5-0.0882 in line 100
- For 50% coincidence, case of equal frequency, use: freq1=0.5+0.1667 in line 96 and freq2=0.5-0.1667 in line 100
- For 10% coincidence, case of equal frequency, use: freq1=0.5+0.0263 in line 96 and freq2=0.5-0.0263 in line 100
- For 50% coincidence, frequencies 2:1 (one input twice more frequent), use: freq1=0.667 + 0.133 in line 96 and freq2=1-0.333 - 0.0667 in line 100
- For 30% coincidence, frequencies 2:1 (one input twice more frequent), use: freq1=0.667 + 0.074 in line 96 and freq2=1-0.333 - 0.037 in line 100
- For frequencies 5:1, 30% coincidence (part P), use: freq1=0.83333 + 0.04386 in line 96 and freq2=1--0.16667-0.00877 in line 100

- Change std as given for the panels you want to reproduce; for that, change values for: std1 and std2 in lines 97 and 101
- Concerning amplitudes, results are calculated and saved for a set of those, see lines 37, 38, 60. Which amplitude to plot is dis-entangled in the plotting program ALL_error_plotting_Fig_6.py

Readout in the plotting program ALL_error_plotting_Fig_6:

- Which amplitude to read-out, change in line 27; relations between indices and amplitudes are given in the comment below. A range of amplitudes can be read out together as given in the provided code
- Raw values (w.o. averaging) can be extracted from variable errors in line 58
- Plotted values end up in errors_matrix (line 63)
- Plotting of that matrix happens in lines 97-110

Figure 7:

First row: BCM_histograms_Fig_7.py

To obtain different panels in in the first row:

- Change amplitude of a second input as required in line 18, variable size
- Change std for both inputs (equal was used) in lines 16 and 20, variables std1 and std2
- For 30% coincidence, case of equal frequency, use: freq1=0.5+0.0882 in line 15 and freq2=0.5-0.0882 in line 19
- For 30% coincidence, frequencies 2:1 (one input twice more frequent), use: freq1=0.667 + 0.074 in line 15 and freq2=1-0.333 - 0.037 in line 19
- Plotting is carried out in the lines 144-147 and numerical values are in the plotted arrays

Second row: Oja_histograms_Fig_7.py

To obtain different panels in in the second row:

- Change amplitude of a second input as required in line 18, variable size
- Change std for both inputs (equal was used) in lines 16 and 20, variables std1 and std2
- For 30% coincidence, case of equal frequency, use: freq1=0.5+0.0882 in line 15 and freq2=0.5-0.0882 in line 19
- For 30% coincidence, frequencies 2:1 (one input twice more frequent), use: freq1=0.667 + 0.074 in line 15 and freq2=1-0.333 - 0.037 in line 19
- Plotting is carried out in the lines 122-125 and numerical values are in the plotted arrays

Third row: Syn_scaling_histograms_Fig_7.py

To obtain different panels in in the third row:

- Change amplitude of a second input as required in line 19, variable size
- Change std for both inputs (equal was used) in lines 16 and 21, variables std1 and std2
- For 30% coincidence, case of equal frequency, use: freq1=0.5+0.0882 in line 15 and freq2=0.5-0.0882 in line 20

- For 30% coincidence, frequencies 2:1 (one input twice more frequent), use: freq1=0.667 + 0.074 in line 15 and freq2=1-0.333 - 0.037 in line 20
- Plotting is carried out in the lines 127-130 and numerical values are in the plotted arrays

Figure 8:

A,B) For ALL rule: ALL_rule_three_inputs_histograms_Figs_8A_and_9A.py

To obtain the plots:

- Set all amplitudes to 1.0, lines 18, 23, 28
- Plotting for (A) is carried out in the lines 189-196 and numerical values are in the plotted arrays
- Plotting for (B) is carried out in the lines 221-223 and numerical values are in the plotted arrays

For BCM rule: BCM_histograms_3inputs_Figure8A.py

To obtain the plots:

- Use parameters as they stand in given code
- Plotting for A is carried out in the lines 149-156 and numerical values are in the plotted arrays
- Run the code several times to randomly obtain different versions provided in the plots
- Plotting for B is carried out in the lines 179-181 and numerical values are in the plotted arrays

C) For ALL rule, first row: run in a sequence:

ALL_error_plots_3inputs_calculation_Fig_8B.py

ALL_error_3inputs_ploting_Fig_8B.py

To calculate different panels in "ALL_error_plots_3inputs_calculation_Fig_8B.py":

- Change amplitudes in lines 80 (size1) and 88 (size 3) to value 1.0; the third amplitude is always kept at value one (size=1.0).
- For changing parameter b, make changes in line 167: f=(sigmoid(10*(outputs_pop1-0.5))-0.1)/0.9; The multiplier in the place …sigmoid(10*… shall be 10, like given, or 5, or 20 for different panels. Sorry, a quick fix here.

To plot in "ALL_error_3inputs_ploting_Fig_8B.py":

- Plotting is carried out in the lines 115-125
- Numerical data is in matrix errors_matrix
- Data before averaging can be extracted from variable errors in line 57

For BCM rule, second row: run in a sequence:

BCM_error_3inputs_calculation_Fig_8B.py

BCM_error_3inputs_plotting_Fig_8B.py

To calculate different panels in "BCM_error_3inputs_calculation_Fig_8B.py":

- Change amplitudes in lines 65 (size1), 70 (size) and 75(size 3) all to value 1.0
- For changing parameter b, make changes in lines
  - 167: f=(sigmoid(10*(outputs_pop1-0.5))-0.1)/0.9 and
  - 153: f_delta=(sigmoid(10*(outputs_pop1+deltay-0.5))-0.1)/0.9

  The multiplier in both expressions the place …sigmoid(10*… shall be 10, like given, or 5, or 20 for different panels. Sorry, a quick fix here.

To plot in "BCM_error_3inputs_plotting_Fig_8B.py ":

- Plotting is carried out in the lines 146-159
- Numerical data is in matrix errors_matrix
- Data before averaging can be extracted from variable errors in line 79

Figure 9:

A) Row 1, error plots: Run in a sequence:

ALL_error_plots_3inputs_calculation_Fig_8B.py

ALL_error_3inputs_ploting_Fig_8B.py

To calculate different panels in "ALL_error_plots_3inputs_calculation_Fig_8B.py":

- Change amplitudes in lines 80 (size1) and 88 (size 3) so that an amplitude set is as indicated above the panel (order does not matter); note, the third amplitude is kept constantly at the value one (size=1).
- Line 167 shall be the following: f=(sigmoid(10*(outputs_pop1-0.5))-0.1)/0.9

To plot in "ALL_error_3inputs_ploting_Fig_8B.py":

- Plotting is carried out in the lines 115-125
- Numerical data is in matrix errors_matrix
- Data before averaging can be extracted from variable errors in line 57

Row 2, example histograms:

ALL_rule_three_inputs_histograms_Figs_8A_and_9A.py

To obtain the plots:

- Set all amplitudes in lines 18, 23, 28 so as indicated in the panels
- Plotting is carried out in the lines 189-196 and numerical values are in the plotted arrays

B) Left, ALL rule: ALL_rule_barplot_5inputs_Figure9B.py

No changes are required to this code.

Right, BCM rule: BCM_barplot_5inputs_Figure_9B.py

No changes are required to this code.

Figure 10:

Left, three inputs: Run a sequence:

command_for_3input_graph_Figure_10_and_11.py (uses graph_3inputs_Figs_10_and_11.py)

combination_analysis_3inputs_graph_Figure_10_11.py

Plot "three_set.pdf" will appear in the working directory

To obtain the plots:

- Run all the codes as they stand
- Plotting is carried out in the lines 147-149 and all values from which the boxplot is calculated can be found in the array zzza

Right, five inputs: Run a sequence:

command_for_5input_graph_Figures_10_and_11.py (uses graph_5inputs_Figs_10_and_11.py)

combination_analysis_5inputs_Figures_10_and_11.py

Plot "thrsets.pdf" will appear in the working directory

To obtain the plots:

- Run all the codes as they stand
- Plotting is carried out in the lines 140-141 of the script " combination_analysis_5inputs_Figures_10_and_11.py"and all values from which the boxplot is calculated can be found in the array zzz created in line 124

Figure 11:

A and B) Three inputs: Run a sequence:

command_for_3input_graph_Figure_10_and_11.py (uses graph_3inputs_Figs_10_and_11.py)

combination_analysis_3inputs_graph_Figure_10_11.py

To obtain the plots in A:

- Change evaluation threshold “thr_eval” in line 61 of the script as required for different column groups in the plot
  “ combination_analysis_3inputs_Figures_10_and_11.py” as required
- Plotting is based on printouts made in line 181. All values can be found in array zzzz formed in line 183

To obtain the plots in B:

- Change variable cell_no in all thre scripts as required (200 or 1000), lines 5, 20, 16 in three scripts. It is redundant.
- Change variable input_no in line 21 of script “graph_3inputs_Figs_10_and_11.py” to 30 in case of cell_no=200 and 150 in case of cell_no=1000
- Change the variables conn_no and conn_center in script “graph_3inputs_Figs_10_and_11.py”, lines 24, 25 to (conn_no =3, conn_center =2) or (conn_no =19, conn_center =10), as required.
- Keep evaluation threshold thr_eval=0.7 in line 61 of the script
  “ combination_analysis_5inputs_Figures_10_and_11.py” as required
- Plotting is based on printouts made in line 181. All values can be found in array zzzz formed in line 183

C and D) Five inputs: Run a sequence

command_for_5input_graph_Figures_10_and_11.py (uses graph_5inputs_Figs_10_and_11.py)

combination_analysis_5inputs_Figures_10_and_11.py

Change in line 88 which threshold to use: pl=copy.deepcopy(pl_07) for threshold 0.7

Printout of the one before last line is put on excel for different cases and makes the figure

To obtain the plots in C:

- Change evaluation threshold by manipulating line 88: pl=copy.deepcopy(pl_07) in script “combination_analysis_5inputs_Figures_10_and_11.py”, where for threshold 0.4 one has to use pl_04 in that line, for threshold 0.5 -> pl_05 and so on.
- Plotting is based on printouts made in line 231 of the script “combination_analysis_5inputs_Figures_10_and_11.py” . All values can be found in array zzzz formed in line 235

To obtain the plots in D:

- Change variable cell_no in all thre scripts as required (200 or 1000), lines 9, 59, 9 in three scripts. It is redundant.
- Change variable input_no in line 60 of script “graph_5inputs_Figs_10_and_11.py” to 30 in case of cell_no=200 and 150 in case of cell_no=1000
- Change the variables conn_no and conn_center in script “graph_5inputs_Figs_10_and_11.py”, lines 63, 64 to (conn_no =3, conn_center =2) or (conn_no =19, conn_center =10), as required.
- Keep evaluation threshold in line 88 as it is: pl=copy.deepcopy(pl_07)

- Plotting is based on printouts made in line 231. All values can be found in array zzzz formed in line 235

Figure 12:

Left, three inputs: run in a sequence:

command_for_3input_graph_Figure_12.py (uses graph_3inputs_Fig_12.py)

combination_analysis_3inputs_graph_Figure_12.py

To obtain the plots:

- Note, first column group is taken from Figure 11, as specified in the paper
- For the following column groups: change in line 16, script "graph_3inputs_Fig_12.py" how much times to increase weights: times_weight_increase=1 (or 1.5 or 2)
- Make sure that line 61 in script "combination_analysis_3inputs_Figure_12.py" reads the following way: thr_eval=0.7.
- Plotting is based on printouts made in line 181 of the script "combination_analysis_3inputs_Figure_12.py" . All values can be found in array zzzz formed in line 183

Right, five inputs: Execute a sequence:

command_for_5input_graph_Figure_12.py (uses graph_5inputs_baseline_Figure_12.py)

combination_analysis_5inputs_Figure_12.py

To obtain the plots:

- Note, first column group is taken from Figure 11, as specified in the paper
- For the following column groups: change in line 56, script "graph_5inputs_baseline_Figure_12.py" how much times to increase weights: times_weight_increase=1 (or 1.5 or 2)
- Make sure that line 89 in script "combination_analysis_5inputs_Figure_12.py" reads the following way: pl=copy.deepcopy(pl_07) (threshold 0.7).
- Plotting is based on printouts made in line 249 of the script "combination_analysis_5inputs_Figure_12.py" . All values can be found in array zzzz formed in line 253

Figure 13:

Run in a sequence:

command_for_5input_graph_Figure_13.py (uses graph_5inputs_inhibition_Figure_13.py)

combination_analysis_5inputs_Figures_13.py

No changes to the codes is needed to obtain the plot provided in the figure. Figure thrsets.pdf (lines 141, 142 in the script “combination_analysis_5inputs_Figures_13.py”) is shown in the paper.

Figure S2.1:

Run in a sequence:

BCM_error_2inputs_calculation_Fig_14.py

BCM_error_2inputs_plotting_Fig_14.py

For Intrator-Cooper version: Line 133 needs be commented out, line 132 shall be un-commented

For Classical BCM: Line 133 shall be un-commented, line 132 shall be commented

Figure “errors.pdf” is given in the paper.
