# Supplementary material for: Unsupervised learning of perceptual feature combinations
Source: PLoS Comput Biol. 2024 Mar 5;20(3):e1011926. doi: 10.1371/journal.pcbi.1011926 (PMC10942261; doi:10.1371/journal.pcbi.1011926)
Supplement: Attachment — Submitted filename: Response_to_reviewers.pdf [file pcbi.1011926.s004.pdf]

Response to reviewers

Thanks to both reviewers for their invested time and for their remarks, even though we have essential objections to the opinion of Reviewer #1, which has led us to file an appeal, which had been granted. We are here, nonetheless, answering also the concerns of Reviewer 1. We have numbered the reviewers' questions/statements and write answers to both reviewers in blue font. We provide line numbers or other pointer to the paper that correspond to our answers, wherever we have made changes.

We also are now submitting code for all result figures as supplementary material. We refined a couple of parameter values in figures and legends, making it consistent with the submitted code.

Reviewer #1:

Q1.
The authors here analyze the general problem of detecting coincident patterns. In particular, they propose a pair of putative learning algorithms based on Hebbian principles with simulated annealing controlling the learning rate and stabilize the output of the cell. This acts to prevent the type o f run-away growth that Hebbian rules often lead to. However, detecting coincident patterns is easily achievable with a Perceptron learning rule and a multi-layer perceptron network. In fact, I would argue that the learning rules they authors consider are essentially slight modifications to the Perceptron learning algorithm where all patterns cause an increase in weights, up until they reach the annealing threshold. As such, the novelty here is minimal as time-varying learning rates in the perceptron learning algorithm are considered even as textbook exercises. It is rather unfortunate, as well, that Perceptrons and the Perceptron learning algorithm is never mentioned in this manuscript, despite the large similarity between both the problem the authors are trying to solve, and the methodology they use. I outline my points below.

We have to object to reviewer's arguments here (and had submitted our appeal on that), as the perceptron learning rule is supervised: it is using an error term between the actual and desired output, while we are proposing an unsupervised rule which without such terms allows for self-organization resulting in neurons signaling coincidences. As we work in the area of unsupervised learning in our paper, we did not cite supervised perceptron learning rule. See more detailed answers below.

Major Concerns

Q2.
1) The specific problem these authors have considered is that of detecting coincident patterns. If we receive multi-modal stimuli that coincide, the neuron should respond but not to the stimuli themselves. If we consider the case of two stimuli, then we can define a binary vector [0,0], [0,1], [1,0], and [1,1] where 1 denotes the presence of a stimulus, and 0 denotes the absence. The goal then is to have a neuron respond exclusively to [1,1]. But, this is accomplished with Frank Rosenblatt's Perceptron learning algorithm, and perceptrons, simple computational units that threshold a linear combination of inputs. Note however that this is a convention. We can equally take the entry "0" to denote the presence of a stimulus, and 1 to denote its absence, or we can define any other vector set (e.g. [-1,-1],[-1,1],[1,-1],[1,1]) to denote the presence or absence of a stimulus.

As we have stated earlier, Rosenblatt's perceptron rule is supervised. Thus, this does not compare to our rule where we work without error terms.

Q3.
2) In fact, I think the authors have largely "rediscovered" the perceptron learning algorithm, and used it somewhat incorrectly.

As Rosenblatt's perceptron learning algorithm is supervised and we are suggesting an unsupervised approach, we find the word "rediscovery" in the above misleading.

Q4.
The author's themselves never cite Rosenblatt's original work, or other perceptron-like pattern classifiers that immediately solve this problem. I outline how the overall setup is largely identical to a perceptron learning algorithm point by point

We did not cite Rosenblatt's work as this work is on supervised classification, while we are considering unsupervised learning approaches in our study and we have concentrated on referencing within this narrower field. However, to reduce confusion, now we write:

> Note, that the weight update routine described above in case of η=0 holds some similarity to Rosenblatt's perceptron learning rule (Rosenblatt, 1958). However, different from Rosenblatt's perceptron, where knowledge on the desired outputs is assumed and error terms are used, we analyze unsupervised learning, where self-organization happens without supplying knowledge about the desired output of the neuron. Thus, these two approaches cannot really be compared.

Q5. (Note, we insert our comments inline within this longer consideration of the reviewer)
- The computational units have a set of inputs u, and a weight vector w. The computational units take the inner product of w with u and then apply some kind of thresholding. This is either smooth, as in the case of the sigmoid considered in equation (2) or discrete, as in equation (7) where y=w^T u must be thresholded by eta. A Perceptron is a computational unit that takes an input pattern x and applies a weight w as an inner product, and subsequently thresholds it:
y = 1 if w^T x >0, 0 if w^T x < 0
- The learning rules are largely perceptron-like. The perceptron learning rule alters the weights as
w(t+delta) = w(t) + r(d_i-y_i)*x_i.
where as the Hebbian rule alters the weight as
w(t+delta)=w(t) + mu(t)*y_i*x_i
and the ALL rule alters the weight as.
w(t+delta) = w(t) + mu(t)*H(y_i-eta)*x_i
where i is the index of the training sample. Let's consider the Hebbian rule, as it is a simpler rule to work with. For all 2D input patterns, [0,0],[0,1],[1,0],[1,1], the ALL-Rule returns the following weight changes
Delta_W = [0,0] when x_i =[0,0]
Delta_W = [0,mu(t)] when x_i = [0,1]
Delta_W = [mu(t),0] when x_i = [1,0]
Delta_W = [mu(t),mu(t)] when x_i = [1,1]
While the Perceptron learning rule would return the following
Delta_W = [0,0]
Delta_W =[0,r(d_i-y_i)]
Delta_W = [r(d_i-y_i),0]
Delta_W = [r(d_i-y_i),r(d_i-y_i)]
Thus, if we consider a thresholded neuron where y_i is in the interval [0,1], and we set d_i = 1 to all the inputs, then we would largely obtain weight changes in a similar direction as the ALL rule. But this would imply all the patterns return a neural activation (y=1).

While the calculations the reviewer is giving may seem related for the two rules, taken one iteration only, there are two issues:

1) d_i in Perceptron rule is the desired output, which is not used in our rule.
2) The reviewer observes himself in the above that if one was using an "arbitrary" signal for the desired output, e.g. d_i=1 all the time, no co-incidence detector would emerge. Thus, this makes it clear that the perceptron rule does not work if the desired output is not given "for real" (i.e., in a differentiated way, where different desired values are given for different input situations)

```
The way the rule seems to work is that you just increase the weight
corresponding to an input generally.  In fact, this is shown in Figure 1.
The weights always increase in response to an input pattern, ti's just the
threshold is set to decrease the learning rate once a neuron fires.   The
coincidence detection is because the two inputs and two positive weights lead
to an overall current that is larger than when just a single input is on.
```

Yes, this is a correct observation of one of the reasons why our rule works, or, stated differently, of the "design" of our rule. We have made Fig. 1. specifically to illustrate that.

```
Normally however, in a Perceptron, you would have negative changes to the
weights which allow you to set a boundary so that not all the responses have
to be +1.
```

Note, whether to use positive or negative changes in the perceptron is determined by the error term. We are not using error terms.

```
The annealing is simply stopping the algorithm from yielding a "spiking" or
threshold response to all possible input vectors, and so you obtain somewhat
coincidentally pattern separation.
```

Note, our paper is made to carry the following message (in layman's terms): if one performs annealing "just in time", that is, when neuron starts to become "active enough" -- this results in coincidence detection. Thus, the statement "`so you obtain somewhat coincidentally pattern separation`" is not correct here. It is our main statement that one can obtain coincidence vs no coincidence pattern separation in this (very simple, unsupervised) way. We clearly state that in the introduction (lines 29 to 43). We have not found this statement made or evaluated in research papers before. Note, we also apply the rule in our paper for recurrent networks, showing that neuronal structures generating possible subsets of a set can be obtained this (simple) way.

Q8.

```
Thus, the algorithms considered by the authors are 1) Perceptron-like in
their deployment, 2) are likely to be less robust than a direct application
of the Perceptron learning rule.   This would also imply that the annealing
threshold is always hand-tuned to guarantee the correct coincident detection.
```

We suggest an unsupervised learning mechanism. It is a trivial fact that, if one adds supervision, it would always allow a method to be more selective and/or robust. However, one needs an error term for that, where the origin of such terms in biological systems is a large unresolved question in its own right. Therefore we are talking about unsupervised mechanisms, which can act in a biological system "as it stands", without the need for the system to "come up" with error terms. Also, note, we have discussed the relation between our method and supervised learning already in our original manuscript, see lines 540-546 in the old manuscript. We now made this discussion point even more explicit by forming a subsection title "Comparison to other learning principles", see lines 634-641 in the new manuscript.

Answering the criticism on “hand tuning”, we have extensively demonstrate robustness of our method in respect to threshold choice and annealing rate (see Figure 6) and this way we, specifically, show that the threshold *does not have* to be accurately hand-tuned for each case. Note, the annealing rate in Figure 6 is given in logarithmic scale, so the areas where coincidence can be detected by ALL-rule in parameter space are really wide.

Minor Concerns

Q9.
1) throughout the manuscripts, the authors have type set the quotation marks wrong. In order to do the left quotation in Latex, one needs to press the ' key twice, while " is used exclusively for the right quotation

Corrected

Q10.
2) This also occurs when using the single quote ' on line 32 for 'large enough'

Corrected

Q11.
3) Munro vs Munroe in the BCM rule, on line 23.

Corrected

Q12.
4) There are many statements made by the authors without supporting references.   For example "annealing is usually applied as an additional mechanism to ensure an efficient convergence of weights"...examples?

We were referring to the preceding sentence which had citations for that. We have now rearranged text to indicate that more clearly. Now we write (lines 48 to 51):

> However, annealing in *those* studies is applied as an additional mechanism to ensure an efficient convergence of weights, while we are not aware of studies which would analyze annealing as the main factor for activity stabilization on its own.

Q13.
5) The figure legends are very spartan at times. For example, for Figure 2, the legend consists of 4 words, with little other description.

Now we extended the legends and also introduced references back to the eqs. the Figure illustrates. Note that Fig. 2 B has been flipped to make the explanations easier.

Q14.
6) I would advise the authors to put their code on a database with a referee read-only access password. I believe github and modeldb have such options.

We now submit our code as zipped file in the supplementary material and will submit the final code to the public Zenodo repository after acceptance of the paper.

Reviewer #2: This article addresses the issue of unsupervised development of an effective coding scheme for feature combinations, an issue that is important for various aspects of cognitive processing.

Thanks for your detailed and – indeed – helpful review. Your consideration about BCM were interesting and we did now dig much deeper into this. Thus, we have now performed many more analyzes. As discussed below (and in the paper), we find that the properties of BCM for variable multiple inputs are complex and lead often to non-desirable and much delayed convergence. The ALL rule performs here better on – essentially – all ends. Please see detailed responses below.

Q1.
In my estimation, the most important element of this article is Figure 7B, bottom-right panel. This is a somewhat non-obvious result, in that it shows that the rule proposed by the authors is potentially capable of generating an ordered representation of feature combinations beyond pairwise, such that it lends itself to easy read-out by subsequent stages. I found it puzzling that this result is relegated to a tiny subpanel within a figure panel, and that it receives little attention beyond this demonstration. The authors should have expanded much more on this result, with more detailed investigations of the minimal conditions that are required for its emergence, its dependence on the number of inputs, parameterization, and others.

In the revised manuscript we added two additional figures showing the results in case of amplitude variation for three inputs (Figure 9A) as well as results in case of five inputs (Figure 9 B). We also have investigated the ranges of annealing parameters allowing to achieve differentiated results for the ALL rule, while showing by the analysis of BCM parameter space that no parameter combination exists where the same could be achieved with the BCM rule (Figure 8C). We lifted results from Figure 7B into Figure 8A to keep all results on ordered representations together. New text on the analysis of ordered representations is provided in lines 379 to 420.

Q2.
As for the remaining part of the article, which forms the bulk of the paper and which focuses almost entirely on pairwise combinations, I was not entirely convinced that it adds much on top of existing schemes. Let's focus on the Intrator-Cooper BCM rule, labelled BCM-rule by the authors. This is the only competing rule selected by the authors that appears as a valid contender to the ALL-rule proposed by the authors. Indeed, except for the result in Figure 7B mentioned above, the BCM-rule is able to accommodate pretty much all other results achieved by the ALL-rule (see for example Figure 7A).

The statement that BCM can do “the same” needs to be taken very much with a grain of salt.

1) Only for simple situations, BCM allows predicting to which fixpoint(s) the weight(s) will converge and for more than 2 variable inputs the fixpoints of BCM do not represent the input characteristics (average amplitude, in our case) any longer. We show this now more clearly by extending the observation that the BCM rule cannot achieve sorting of coincidences with 3 or 5 inputs (see figures 8 and 9), while the ALL rule can.

2) Furthermore, note the very much delayed convergence of the BCM rule in case of multiple inputs discussed by Froc and v. Rossum (2019). We observe the same unwanted property of BCM in our study (see panel B in Figure 8), in case of three or more inputs, and we remark on it now (lines 370-378 and 599-607) and show converged plots in the case of three inputs after 100,000 iterations, where for five inputs we sometimes found far longer learning periods (up to 2 million iterations!). From a biophysical perspective such high numbers are not compatible with any learning success during the lifetime of an animal. The ALL rule converges after about 100 iterations and is not much affected by the number of inputs.

3) In addition, one can observe that substantial reordering of the weights can occur late in learning (especially in cases where v_0 is small). Hence, there is not even an approximate steady state assured until final (late) convergence.

4) The literature discusses several versions of the BCM rule. The more advanced versions have – indeed – more advantageous computational properties than the original rule. They are, however, reaching a level of complexity which is not any longer easily compatible with the biophysics of neurons (more details in response to Q4). By contrast, the ALL rule is far simpler, which is another advantage.

5) For the interested reader, we now also include results on robustness of BCM rule to parameter changes in the Appendix, where we show that the BCM rule in the context of the task with two inputs (using the classical formulation as well as the BCM-Intrator-Cooper formulation) is sensitive to parameter v_0, also in the two input case. Interpretation of v_0 is that of being a "target value" of the neuron in case of standard BCM without non-linearity in the output and when only one constant input is given to a neuron (Toyoizumi et, al, 2014). However, this parameter does not have such a clear meaning in case of several variable inputs. Versions of the rule without v_0 (i.e., v_0=1, Blais & Cooper, 2008) would not work for the task we are presenting here at all.

Q3.

When I look at equation 9 (BCM-rule), it seems plausible that it may be made to behave similarly to equation 3 (ALL-rule). \mu and \mathbf{u} are common to both rules. We are left with a bunch of stuff in equation 9 that depends on v, where v is a function of the linear output y, corresponding to g in equation 3, which is a thresholded (Heaviside) function of y. As noted by the authors, v can be made to approximate the thresholding function in equation 3 (lines 127-128), so the question is whether v^2*dv/dy (with \Theta_{M} set to ~0) may behave like v when v is very sharp, which is not implausible depending on details. I may have missed it, but I could not find a detailed description of the parameterization adopted for the BCM-rule, so I assume that the authors chose a parameterization for v that is quite gradual. I wonder how the BCM-rule behaves for the specific case shown in Figure 7B when it is parameterized to implement a sharp thresholding function v. It may produce a better separation for the triple conjunction, for example.

There are 3 points to answer here:

1) The parameterization for v used in the BCM rule was the same as for all other rules in the paper with parameters given in equation (2) and the function visualized in Figure 2.

2) Concerning making the behavior of BCM similar to our rule in case of "very sharp v" (with \Theta_{M} set to ~0): Note that, first, if we set \Theta_{M} to ~0 the resulting rule will then be quite different from the BCM rule. In the BCM rule, the term \Theta_{M}, by the design of the rule, depends on v (that is, it is not a free parameter) and acts as a variable threshold assuring convergence. If we set \Theta_M to zero, convergence of the rule would be assured for either v=0 or dv/dy=0. Commonly one uses a sigmoidal activation function (such as in our case, too). Then, the second condition is reached only at infinity. Thus, one gets a system with asymptotic convergence as shown in the figure below (three weights: blue, yellow and green approaching an asymptote at infinity):

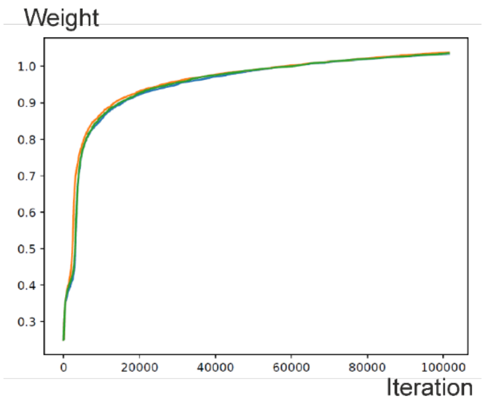

Such a behavior, however, is not compatible with the tasks addressed in this study and the thus-resulting rule is anyhow rather far away from BCM.

3) Concerning the suggestion that the BCM rule with sharp thresholding function v could produce better separation of triplets: we analyzed the BCM and ALL rules with different steepness of v in Figure 8 now. While the ALL rule solves the task for a large range of parameters for different-sharp v, the BCM rule cannot solve the task neither by tuning parameter v_0, nor by changing the v-sharpness (second row of plots in Figure 8B).

Q4.
If the Intrator-Cooper BCM rule can be parameterized to encompass the results achieved by the ALL-rule, it is still interesting to show that it can be significantly simplified to acquire unexpected properties like Figure 7 (bottom-right panel), but it does subtract somewhat from the contribution of this study in terms of novelty. The issue here is that the ALL-rule may bring some benefit in relation to a specific problem like the one examined here, but may underperform with respect to a different problem. If the BCM-rule can potentially cover the ALL-rule and also deal with other problems, it would remain a more attractive option.

As indicated in the answer above (Q3), the BCM rule cannot perform the input sorting task. As it is also argued in answer to Q2, the BCM rule with more than two inputs tends to have a quite delayed convergence.

The reviewer rightfully notices, that the ALL rule is much simpler than the BCM rule. Now we emphasize the simplicity of the ALL rule in the paper even stronger. Simplicity of a rule is an important aspect, considering possible biological mechanisms to implement the rule and also (possibly) concerning energy efficiency when such a rule is operating. BCM requires here far more effort than ALL and – also different from ALL – not all operations of BCM are easily compatible with a biophysically realistic implementation. For example, the calculation of v^2*dv/dy appears biophysically quite difficult. We remark on this in the discussion now, lines 608-611

Q5.
Another (minor) criticism is that the authors choose relatively narrow ranges for input parameterization, for example they explore two inputs that differ in intensity by about 50%, which is not very useful for real biological processing: under real-world scenarios, features must be combined under much more extreme changes in input intensity. I realize that the approach can be augmented to incorporate normalization schemes that act early, but it is not then clear that the ALL-rule would preserve the ordered structure demonstrated in Figure 7B under those conditions. I think this aspect of the study needs further investigation/clarification.

Please see answer to Q6 where will address this issue, too.

Q6.
An issue related to the one above is that, possibly as a consequence of the limited amount of details provided by the authors in relation to the training procedure, it was not clear to me how robust the ALL-rule is to potential differences between the training cohort and a subsequent testing cohort that differs significantly, particularly in relation to coding stability. For example, once the network is frozen, is it stable when coding input distributions that differ from those used during training, and that differ among themselves? By stable I mean that if a given neuron codes for combination 13 or 123, it retains its label regardless of substantial changes in the parameterization of the input. For example, if input 1 was twice as intense as input 2 during training, and the network is characterized for this configuration, when I then characterize for a configuration in which input 1 is half as intense as input 2, is the code stable? These issues do not seem to be adequately
addressed.

1) Concerning Q5: Large amplitude differences will indeed harm performance. However, there is strong evidence that normalization is a powerful mechanism in many different brain areas (see review by Carandini and Heeger, 2012). Note that a factor of 1.5 – like for the amplitudes in our experiments – is clearly within the normalization regimes for many of these experimental findings. This is now better discussed (lines 658-666).

2) Furthermore, note that in Figure 6 panel P we have investigated a case of a five-fold difference in occurrence frequency and an amplitude difference of 1.5. Hence, one stimulus *intensity* can be 7.5 times bigger than the other and errors are still limited. Hence, the system tolerates a reasonable degree of amplitude variability mixed with a large degree of stimulus-occurrence frequency difference. In an ecological setting animals have no control over the latter, where by normalization mechanisms negative effects of the former can be ameliorated.

3) Concerning Q6: We now emphasize more strongly that we are analyzing a stationary environment (see lines 644-645 in the "Limitations" section). Thus, the basic assumption is that there is little or no change between statistics of training and testing samples. However, as by response (2) above, please note, that we are already showing results in case of relatively wide input amplitude as well as frequency distributions (large differences paired with relatively large std). This demonstrates that the ALL-system is to a useful degree robust against "changes in the environment".

4) We also discuss that the issue of a non-stationary environment can possible by addressed by weight-decay mechanisms (lines 645-649). In view of the very fast convergence of the ALL rule, weight-readjustments to a changed environment can this way commence quickly. However, we think that this is a matter of future investigation as this paper is by now already quite complex.

Have the authors made all data and (if applicable) computational code underlying the findings in their manuscript fully available?
The PLOS Data policy requires authors to make all data and code underlying the findings described in their manuscript fully available without restriction, with rare exception (please refer to the Data Availability Statement in the manuscript PDF file). The data and code should be provided as part of the manuscript or its supporting information, or deposited to a public repository. For example, in addition to summary statistics, the data points behind means, medians and variance measures should be available. If there are restrictions on publicly sharing data or

code —e.g. participant privacy or use of data from a third party—those must be specified.

Reviewer #1: No: The authors won't make their code public until after the manuscript has been accepted, but have also not included links for a referee.

Reviewer #2: None

Reviewer #2: No

Note, now we have submitted the code as a zip file in the supplementary material and will be in addition submitting it to a public repository after acceptance of the paper.

References

Brian S. Blais and Leon Cooper (2008) BCM theory. Scholarpedia, 3(3):1570.

Carandini, M., & Heeger, D. J. (2012). Normalization as a canonical neural computation. *Nature Reviews Neuroscience*, *13*(1), 51-62.

Froc, M., & van Rossum, M. C. (2019). Slowdown of BCM plasticity with many synapses. *Journal of Computational Neuroscience*, *46*, 141-144.

Rosenblatt, F. (1958). The perceptron: a probabilistic model for information storage and organization in the brain. *Psychological review*, *65*(6), 386.

Toyoizumi, T., Kaneko, M., Stryker, M. P., & Miller, K. D. (2014). Modeling the dynamic interaction of Hebbian and homeostatic plasticity. *Neuron*, *84*(2), 497-510.
