# Supplementary material for: Unsupervised learning of perceptual feature combinations
Source: PLoS Comput Biol. 2024 Mar 5;20(3):e1011926. doi: 10.1371/journal.pcbi.1011926 (PMC10942261; doi:10.1371/journal.pcbi.1011926)
Supplement: Attachment — Submitted filename: Responses_to_reviewer_comments_revision2.pdf [file pcbi.1011926.s005.pdf]

Thanks to the reviewers and to the editor for positively evaluating our review work!

In the following text we will write our answers in blue font, Calibri.

Answers to Editor

Thank you very much for submitting your manuscript "Unsupervised learning of perceptual feature combinations" for consideration at PLOS Computational Biology. As with all papers reviewed by the journal, your manuscript was reviewed by members of the editorial board and by several independent reviewers. The reviewers appreciated the attention to an important topic. Based on the reviews, we are likely to accept this manuscript for publication, providing that you modify the manuscript according to the review recommendations.

Thank you for positive evaluation!

In particular, the authors should do a little more to clarify the distinction between their unsupervised learning rule and the Perceptron rule, as well as the advantages of taking an unsupervised approach (i.e. biological plausibility, no need for feedback). However, I do not think they need to formally compare the results obtained using each approach.

Done, see replies to reviewer 3 and blue-marked changed in the ms.

When you are ready to resubmit, please upload the following:

[1] A letter containing a detailed list of your responses to all review comments, and a description of the changes you have made in the manuscript. Please note while forming your response, if your article is accepted, you may have the opportunity to make the peer review history publicly available. The record will include editor decision letters (with reviews) and your responses to reviewer comments. If eligible, we will contact you to opt in or out

[2] Two versions of the revised manuscript: one with either highlights or tracked changes denoting where the text has been changed; the other a clean version (uploaded as the manuscript file).

The indicated files have been uploaded to the system.

We have also updated our figures using PACE tool and uploaded a new version.

We have also expanded our “readme” file for the submitted computer code, so that each panel of each figure in the manuscript can be reproduced with ease.

Answers to reviewers

Reviewer #2:

I should state upfront that, in general, I do not like to re-review rejected papers. This kind of situation makes peer review messy. Having said that, I sympathize with the authors in that they seem to have reasonable grounds for appeal in this case.

Thanks for your understanding!

I should also notice upfront that, when going back to this manuscript, I was irritated by the fact that the authors did not highlight revisions using, for example, red ink. This means that Reviewers must re-read the paper from scratch, which adds to their workload (and I am reviewing another 4 papers on top of this one). In the future, please be more respectful of Reviewers' time by clearly highlighting portions of the manuscript that have been modified during the revision process.

This must have been some miss-understanding, as the Journal requires to submit both, the non-highlight and the highlighted version and we did that.

Now, with all that in mind, I am trying to keep an open mind here. The authors have expanded on the feature I singled out during the first round, namely the result in Figure 7 that was originally relegated to one subpanel. In my opinion, that is the entire paper: without that, I would not consider this manuscript above threshold for PLOS CB. With that result, I think it deserves consideration. In my understanding, it is an interesting result that such a simple rule can generate an ordered representation of feature combinations that is essentially transparent for further read-out by higher modules.

Thank you very much for the open mind and for the positive evaluation!

I was somewhat disappointed by the fact that the authors did not take on the challenge I set out for them in the first round regarding stability of the coding strategy (point they refer to as Q6 in their rebuttal). At the same time, I accept that this is a very challenging problem, so I am willing to concede on that point and accept that it will require further research beyond this paper to be fully addressed.

Thanks, one more time!

All in all, the authors have addressed my comments to a reasonable degree of satisfaction.

Reviewer #3:

I will not provide a detailed review of this paper, as I was brought in after an initial round of reviews had already taken place. Instead, I will focus primarily on the dispute/appeal regarding

reviewer 1's comments on the manuscript. Having read through the reviewer comments and author responses, I am satisfied that the author's are correct in their response, and that the reviewer's objection was based on a misconception/failure to distinguish between supervised/unsupervised learning rules. That said, the very fact that the reviewer (who is presumably an expert in the field) was able to come to this erroneous conclusion suggests that the authors have not taken enough care to clarify the essential contributions of their study and the relevance to previous work. Therefore, I would recommend that the authors explicitly discuss the distinction between supervised/unsupervised learning rules, and make clear to the reader how their work goes beyond simple perceptron learning rules/is more biologically plausible, etc. (it is of course up to the authors to decide how to frame the contributions of their paper).

To better inform the reader, we have now strengthened the claim in the introduction that we investigate unsupervised learning. Now we write (old writing in black, updates blue):

> *…A central problem that arises here is that features will not only occur in combination but also on their own. This will happen with different individual- as well as coincidence-occurrence frequencies and, in addition, usually also with variable intensity. Thus, in order to learn the meaning of combined features, the nervous system must learn this without being distracted by this variability. While supervised learning methods, like the LMS algorithm (Widrow & Hoff, 1960), could address this problem in efficient way, here we investigate unsupervised learning. The latter is much simpler from the point of view of biological implementation, as it does not require additional evaluative sub-systems and mechanisms. As shown below, with unsupervised learning one can detect feature combinations already at the level of a single neuron. To achieve this, neuronal plasticity must come to a halt as soon as a combination has been recognized, otherwise ongoing plasticity would lead to undesired responses to individual features.*

While the authors have added a small paragraph attempting to address this, I found this unsatisfactory and somewhat dismissive of reviewer 1's concerns (e.g., "these two approaches cannot really be compared"). Instead, the authors should explain why this distinction/advance is important, and why this makes the proposed learning rule advantageous. Even better would be if the authors could formally (via numerical simulations and/or mathematical analysis) compare and contrast the supervised perceptron learning rule vs their method (of course supervised will do better, and that's not a problem, but some general insight into how the two learning rules relate could be interesting). Overall, the comparison of this learning rule to the classic perceptron learning rule seems important, as has not been sufficiently elaborated in the manuscript as far as I can tell.

We have lifted the comparison of the supervised and unsupervised rules into the discussion section. Now we write (old writing in black, new in blue):

> *Clearly, reinforcement learning and supervised learning would be able to achieve the tasks investigated in this study, too. Both, however, require evaluative feedback in the form of rewards or by use of an error function. While evaluative feedback can help achieving more discriminative results in learning tasks, not excluding the here-addressed task of coincidence sorting, the origin of error terms in biological systems is a*

large unresolved question in its own right (Burton & Balleine, 2022) . We had discussed that there are formal similarities between our rule (eq. 8}) and Rosenblatt's perceptron (Rosenblatt,1958), but for the perceptron an error term is needed. Note, that error terms in biological systems do not come ``for free''. Any system using evaluative feedback needs additional components and complex processes. The multifaceted properties of the dopaminergic system in the animal brain (i.e. a reward-processing system which, however, also strongly reacts to just a novelty signal) testifies to this complexity (Glimcher, 2011, Duszkiewicz et al, 2019). Different from this, our method is non-evaluative and performs a process of self-organized stimulus sorting in a single neuron. Any potential ecologically meaningful evaluation could then come on top and, for example, reinforcement learning of a beneficial behavioral policy could make use of the responses of our feature-combination specific neurons.

Concerning numerical simulations of Rosenblatt's rule: as we state now in the introduction, we are interested in the self-organization of feature combination detection in a small neural system (one neuron), and therefore we designed and analyzed our unsupervised learning rule for that. Thus, numerical comparison of the suggested rule to algorithms of supervised co-incidence detection is not in the core of this study. Here we also follow the editor's suggestion that a formal comparison on results obtained using each approach is not needed.

References

1. Burton TJ, Balleine BW. The positive valence system, adaptive behaviour and the origins of reward. Emerging Topics in Life Sciences. 2022;6(5):501–513.
2. Duszkiewicz AJ, McNamara CG, Takeuchi T, Genzel L. Novelty and dopaminergic modulation of memory persistence: a tale of two systems. Trends in neurosciences. 2019;42(2):102–114.
3. Glimcher PW. Understanding dopamine and reinforcement learning: the dopamine reward prediction error hypothesis. Proceedings of the National Academy of Sciences. 2011;108(supplement 3):15647–15654
4. Rosenblatt F. The perceptron: a probabilistic model for information storage and organization in the brain. Psychological review. 1958;65(6):386.
5. Widrow B, Hoff ME. Adaptive switching circuits. In: 1960 IRE WESCON Convention Record, Part 4. New York; 1960. p. 96–104

Have the authors made all data and (if applicable) computational code underlying the findings in their manuscript fully available?

The PLOS Data policy requires authors to make all data and code underlying the findings described in their manuscript fully available without restriction, with rare exception (please refer to the Data Availability Statement in the manuscript PDF file). The data and code should be provided as part of the manuscript or its supporting information, or deposited to a public repository. For example, in addition to summary statistics, the data points behind means, medians and variance measures should be available. If there are restrictions on publicly sharing data or code —e.g. participant privacy or use of data from a third party—those must be specified.

Reviewer #2: Yes

Reviewer #3: Yes
